# Osteoid Osteoma of the Hand: Surgical Treatment versus CT-Guided Percutaneous Radiofrequency Thermal Ablation

**DOI:** 10.3390/life13061351

**Published:** 2023-06-08

**Authors:** Fabio Vita, Gianmarco Tuzzato, Davide Pederiva, Giuseppe Bianchi, Augusto Marcuzzi, Roberto Adani, Paolo Spinnato, Marco Miceli, Danilo Donati, Marco Manzetti, Federico Pilla, Cesare Faldini

**Affiliations:** 1 IRCCS-Rizzoli Orthopedic Institute, University of Bologna, 40136 Bologna, Italy; gianmarco.tuzzato@ior.it (G.T.); davide.pederiva@ior.it (D.P.); giuseppe.bianchi@ior.it (G.B.); paolo.spinnato@ior.it (P.S.); miceli.marco@aou.mo.it (M.M.); marco.manzetti@ior.it (M.M.); federico.pilla@ior.it (F.P.); cesare.faldini@ior.it (C.F.); 2 Department of Hand Surgery and Microsurgery, University Hospital of Modena, 41124 Modena, Italyadani.roberto@aou.mo.it (R.A.); da.donati@yahoo.it (D.D.)

**Keywords:** osteoid osteoma, hand, benign tumor, radiofrequency thermal ablation

## Abstract

(1) Background: Osteoid osteoma (OO) is one of the most common benign bone tumors. This type of osteogenic tumor is generally characterized by a well-defined lytic area with a vascularized central nidus surrounded by sclerosis and bone thickening. The wrist and hand bones are infrequent sites for osteoid osteoma: only 10% of the cases arise in these areas. Standard treatments are surgical excision and radio-frequency ablation (RFA), both with advantages and disadvantages. This study aimed to compare the two techniques to prove if RFA could be a potential alternative to surgery in the treatment of OO of the hand. (2) Methods: Patients treated for OO of the hand between January 2011 and December 2020 were evaluated and data was collected regarding the lesions’ characteristics and the treatment outcome. Each patient was followed up for 24 months and VAS pain (Visual Analogue Scale), DASH (Disability of the Arm, Shoulder and Hand), and PRWE (Patient-Related Wrist Evaluation) scores were collected. (3) Results: A total of 27 patients were included in the study: 19 surgical and 8 RFA. Both treatments showed a significant improvement in pain and functionality. Surgery was associated with a higher complication rate (stiffness and pain), while RFA was associated with a higher recurrence rate (2/8 patients). RFA allowed for a speedier return to work. (4) Conclusions: We believe that osteoid osteoma treatment with RFA in the hand should be an available alternative to surgery as it allows rapid pain relief and a swift return to work. Surgery should be reserved for cases of diagnostic uncertainty or periosteal localization.

## 1. Introduction

Besides enchondroma, osteoid osteoma (OO) is one of the most common benign bone tumors. This type of osteogenic tumor is generally characterized by a well-defined lytic area with a vascularized central nidus surrounded by sclerosis and bone thickening [1]. The lesion typically occurs in children and young adults between the first and third decades of life and is usually localized in the long bones of the lower limbs [2]. The most characteristic symptom is nocturnal pain, which is usually relieved by non-steroidal anti-inflammatory drugs (NSAIDs) [3].

Wrist and hand bones are rare sites for osteoid osteoma: only 10% of cases occur in these areas [4]. Minimally invasive techniques such as radio-frequency ablation (RFA) [5], microwave ablation, or non-invasive ones such as Magnetic Resonance Imaging guided Focused Ultrasound (FUS) are nowadays considered the first-line treatments option for this condition [6]. Among those, RFA is still the most commonly used method with proven efficacy [7,8].

Surgery is considered the treatment of choice in areas where thermal ablations carry the risk of nerve or skin damage, especially in some spinal locations [9]. Surgery is still considered the gold standard because it provides direct visualization for complete removal of the mass. However, surgical intervention is not without complications such as fractures and scar adhesions and has a greater difficulty in reaching and identifying the small nidus when is deeply located [5,10].

RFA, on the other hand, is a minimally invasive procedure. An electrode connected to a radio-frequency generator is inserted into the nidus of the tumor guided by a CT scan and, by heating the lesion, induces tissue necrosis [11]. This approach involves minimal tissue trauma and lower cost and also requires a shorter recovery time and hospital stay. The major limitation with this technique is that the high generated temperature may damage sensitive tissues such as skin and nerves if the lesions are smaller than 1 cm. For this reason, osteoid osteoma of the hand is rarely treated with RFA [12,13].

We believe that a comprehensive knowledge of the pathology and the used instruments allows for the safe and efficient use of RFA in the treatment of neoformations of the hand. This study was designed to compare surgical treatment with radio-frequency ablation for the treatment of osteoid osteoma in the hand.

## 2. Materials and Methods

A retrospective analysis was conducted on patients with OO of the hand that underwent surgical excision between January 2011 and December 2017 and radiofrequency ablation between January 2018 and December 2020. Inclusion criteria were the presence of clinical symptoms such as local pain in the hand, relief after the use of anti-inflammatory drugs, and a radiologic clear evidence of osteoid osteoma [1]. Patients with previous surgical or percutaneous treatment or that had initially been managed by other centers (*n* = 8) were excluded from the study.

A total of 27 patients were enrolled in the study. Of these patients the following were recorded: age, sex, type of work (heavy manual labor, light office labor, or unemployed), the side affected by the disease, site (carpal, metacarpal, phalanges), bone placement (periosteal, intracortical, subcortical) and size (<5 mm or ≥5 mm, according to the largest diameter of the nidus seen at the CT scan [1]) of the disease. Patients were then divided according to treatment choice (surgical excision or radiofrequency ablation) and the following were recorded: complications such as stiffness, pain, intraoperative fracture, local relapse (intended as the failure of completely removing the neoformation), heat lesions (intended as damage to soft tissues due to the heat generated by the RFA electrode); recurrence (intended as the reappearance of the symptoms after a disease-free period of at least 3 months); and return to work recorded as the number of weeks required to resume full work activity.

Patients were treated with surgical excision from January 2011 until December 2017. From January 2018 radiofrequency ablation was implemented as the treatment of choice for OO of the hand. According to the literature [14], periosteal OOs encountered after January 2018 were treated surgically, and, as this position, being too superficial and within proximity to critical soft tissue structures, represents a contraindication to RFA, it was therefore excluded from the study to avoid creating a timeline–treatment overlap.

**Figure 1:**
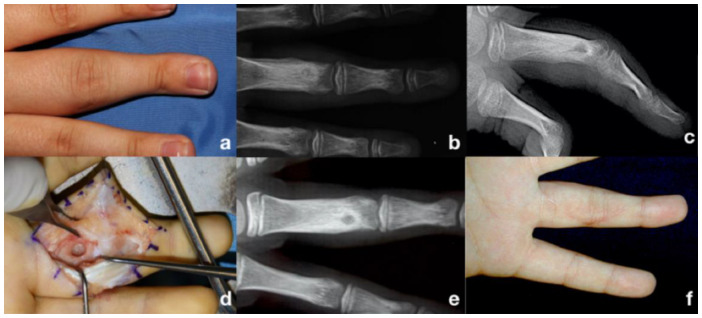
Osteoid osteoma in the proximal phalanx of the 3rd finger. (**a**) Preoperative clinical picture. (**b**) Preoperative anteroposterior XR. (**c**) Preoperative laterolateral XR. (**d**) Surgical access and OO removal after bone curettage. (**e**) Postoperative anteroposterior XR. (**f**) The postoperative clinical picture at 1 month of follow-up.

The surgical technique consisted of bone curettage of the lesion using a local anesthetic (Figure 1). The diagnosis was confirmed by histopathological examination [15]. The use of autologous bone graft was evaluated case by case according to the intraoperative evaluated risk of following postoperative fracture [16]. Only lesions bigger than 5 mm were filled with bone grafts, collected either from the distal radius or from the iliac crest according to the performing surgeon’s preference.

As for the radiofrequency ablation, with the use of local anesthesia, the procedure was performed using a CT-guided percutaneous needle [2]. Before the treatment, the lesion was drilled introducing a needle for a bone biopsy, which was used to confirm the diagnosis histologically. Then, the 5 mm RFA electrode was inserted into the bone tunnel checking with CT scan the correct position within the center of the nidus, thus preventing skin and soft tissue burns. The electrode was then connected to a generator reaching the temperature of 75 °C for 4 min [13,17], causing bone necrosis in a spherical area of approximately 1 cm in diameter [18].

After the treatment, patients were discharged home within 6–8 h with a prescription of analgesic drugs and without special restrictions of movement or physical activity. Return to work was decided on a case-by-case basis in light of the clinical picture. Follow-up visits were performed at 1, 3, 6, and 24 months comprising a clinical and X-ray evaluation.

Each patient was assessed with a VAS score for pain (Visual Analog Scale: 0 no pain, 10 maximum pain), while the functional outcomes were measured according to validated scores such as the DASH score (Disability of the Arm, Shoulder, and Hand, out of 100 points) [19] and the PRWE score (Patient Rated Wrist/Hand evaluation, grades from 0 to 100 points) [20]. These scores were recorded pre-operatively and at each follow-up visit. The pre-operative scores were compared to the ones collected at the last follow-up visit to seek any statistically significant difference.

The study was conducted in accordance with the ethical standards of the Declaration of Helsinki and informed consent was obtained from all patients. Statistical analysis was performed using the Kruskal–Wallis test and the Fisher’s exact test. Statistical significance was set at a *p*-value < 0.05. Statistical analysis was performed using GraphPad Prism (version 8.0, GraphPad Software, La Jolla, CA, USA).

## 3. Results

Between January 2011 and December 2020, a total of 27 patients were treated for OO of the hand either with surgical excision or with radiofrequency ablation and thus enrolled in the study.

**Figure 2:**
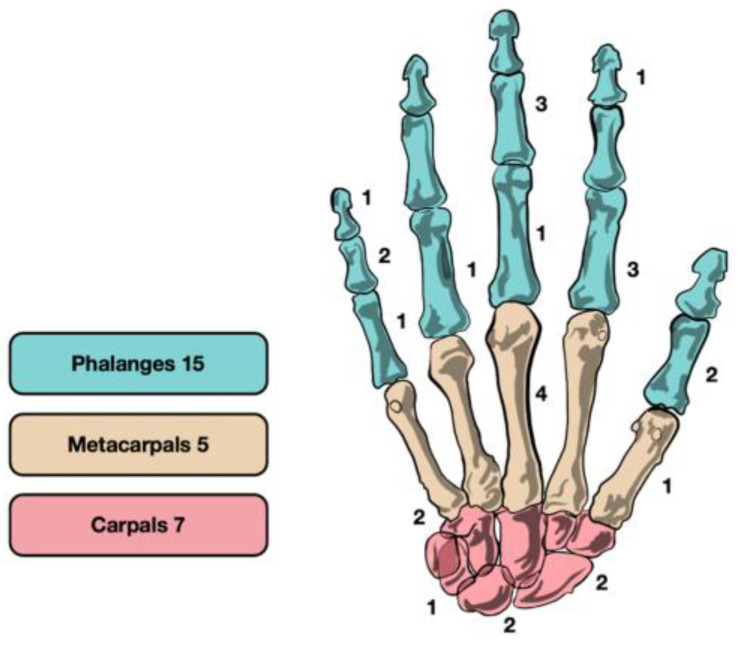
The overall distribution of osteoid osteoma in the hand.

The mean age at the time of surgery was of 30.8 years (range 12–59): 16 patients were male (59.3%) and 11 were female (40.7%). The right hand was affected in 15 (55.6%) cases and the left in 12 (44.4%) cases. Further, 15 of the osteoid osteomas (55.6%) were located in the phalanges, while 5 (18.5%) were found in the metacarpals and 7 (25.9%) in the carpal bones (2 scaphoid, 2 lunate, 2 hamate, and 1 triquetrum) (Figure 2). In 12 cases (44.4%), the osteoid osteoma was intracortical, in 7 patients (25.9%) it was periosteal, and in 8 (29.6%) cases subcortical. General characteristics are summarized in Table 1.

A total of 19 patients (8 male, 11 female) were treated by surgical approach between January 2011 and December 2017 (Table 2). The mean age was 30.2 years and 47.4% (9/19) of them were heavy manual workers.

**Figure 3:**
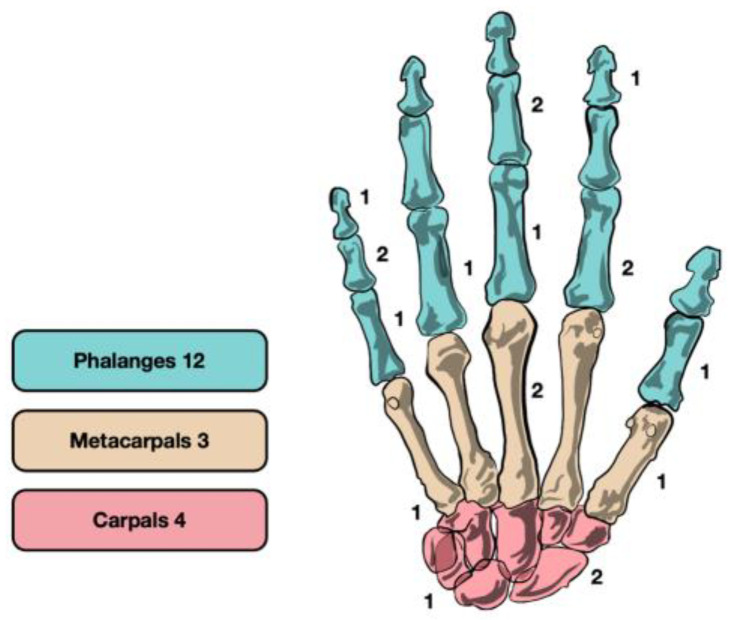
Surgical group distribution of osteoid osteoma.

The lesions were mainly localized in the phalanges (12/19–63.2%), followed by carpal bones (4/19–21.0%), and metacarpals (3/19–15.8%) (Figure 3). No predominant cortical relation was recorded (7 periosteal, 8 intracortical, 4 subcortical), and 8 lesions (42.1%) were bigger than 5 mm and were filled with autologous bone graft (5 collected from the distal radius, 3 from the iliac crest).

Following surgery, 6 patients (31.6%) experienced complications: 1 reported stiffness in finger movement at the proximal interphalangeal joint (improved only partially with physiotherapy), 3 reported moderate pain at the excision site, 1 intraoperative iatrogenic fracture was observed (a finger splint was placed and kept for 4 weeks), and 1 local relapse was recorded (a second operation was performed at 6 months from the first one with symptoms resolution). There was no recurrence of the disease. Return to work was at 3 weeks for 15 (79.0%) patients and was delayed to 5 weeks in 4 (21.0%) patients because of complications (3 because of persistent pain and 1 because of intraoperative fracture).

A total of 8 patients (3 male, 5 female) underwent radiofrequency ablation from January 2018 to December 2020 (Table 2). The mean age was 31.7 years and 50.0% (4/8) of them were heavy manual workers.

**Figure 4:**
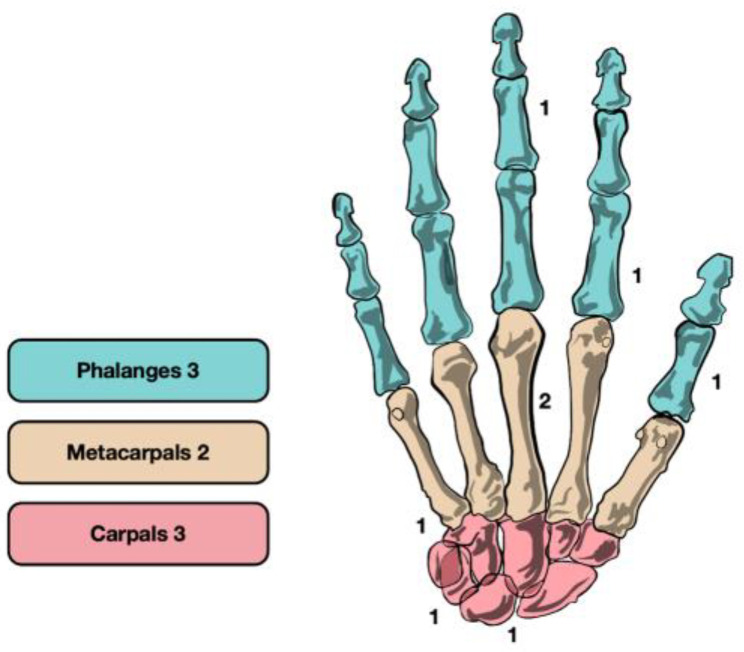
RFA group distribution of osteoid osteoma.

The lesions did not show a predominant localization (3 phalanges, 2 metacarpal, 3 carpal) (Figure 4). Of the 8 patients treated by RFA, 4 (50.0%) had intracortical lesions and 4 (50.0%) had subcortical lesions. No periosteal OO was treated by RFA, for the reasons stated above. Of the 8 lesions treated, 6 (75.0%) were bigger than 5 mm.

Following the procedure there was only 1 (12.5%) complication: a local relapse (a second ablation was performed 3 months later with resolution of symptoms). No patient experienced heat lesions, paresthesia, or stiffness. During the follow-up a recurrence was recorded at 6 months, the OO was localized in the proximal phalanx intracortical with a diameter of 6 mm, and the patient was treated again with RFA with a successful outcome. Return to work was at 2 weeks for every patient.

Before treatment, all patients with OO reported clinical manifestations such as mild limitations of daily manual activities, swelling, and nocturnal pain with a median duration of 6 months.

Both surgery and RFA were able to reduce VAS pain (from 7 to 2 and from 8 to 1, respectively) and improve both DASH score (from 40 to 18 and from 35 to 19, respectively) and PRWE score (from 56 to 22 and from 50 to 18, respectively) from pre-operative to the last follow-up visit. The improvement was statistically and clinically significant (Table 3).

Of the 19 patients treated with surgery, 11 were completely painless, while 8 patients occasionally presented mild pain (VAS 1-2); of the 8 patients treated with RFA, 6 were completely painless, while 2 patients had occasional mild pain (VAS 1). No patient treated described limitations on daily activities, and all but two patients (92.6%) were satisfied or very satisfied with the treatment.

## 4. Discussion

Osteoid osteoma is a common benign lesion of the bone; it is typically found in the lower extremities and is only rarely detected in the hand [21,22]. Our study aimed at discussing two possible treatments: surgical excision by curettage with or without bone grafting and radiofrequency thermal ablation. Both treatments showed promising results with an improvement both in pain and in function.

Curettage is currently the most used technique. Emptying the bone causes a reduction in its structural strength. Grafts, autologous or allogeneic, can be used to reduce the risk of postoperative fractures. The use of graft-associated curettage does not expose to a greater risk of complications or local recurrence [23]. Several complications have, however, been found associated with surgery: rigidity, pain, and fractures [5,10]. To reduce these scenarios radiofrequency ablation was proposed as a form of treatment [24].

RFA treatment is mainly used in different skeletal segments, more difficult to reach surgically [8,13]. The first treatment reported with RFA was published in 1992 by Rosenthal [25]. Since then, RFA treatment, carried out in most bones of the body, has been widely used for years. Rosenthal et al. treated 33 patients with osteoid osteoma in several different locations (femur, tibia, humerus, coracoid, acetabulum, radius) with an average follow-up of 9 years and a recurrence rate of 12% [26]. Woertler et al. treated 47 patients with RFA with a follow-up of 22 months and a recurrence rate of 4.2%, treated with success with a second ablation [17]. Assoun et al. [27] reported successful RFA treatment for 96% of cases in a study of 24 patients, while Sans et al. [28] reported a successful RFA treatment in 88% of cases for a group of 38 patients. Such studies prove that our RFA recurrence rate of 12.5% is in line with the literature, and we believe that a larger sample size would probably lead to a reduction in this value.

Because RFA treatment causes necrosis in an area of 1 cm in diameter, many authors have refused to treat the osteoid osteoma of the hand afraid of the risk of iatrogenic soft tissue damage [13]. To reduce these possible complications recent studies have suggested reducing the generated heat from 90 °C to 75 °C [13,17,29], just as we did with optimal results. Another suggested technique to reduce skin burns and superficial soft tissue heat lesions is the application of a gauze soaked in cold sterile saline covering the entry point of the electrode [24]. By doing so the damage to neighboring tissues is greatly reduced, widening the possible therapeutic indications. However, this risk is sometimes inevitable, and it needs to be discussed with the patients beforehand.

The evidence of the use of RFA in the hand is still limited. Bailey et al. [30] successfully treated an osteoid osteoma of a second metacarpal in a 20-year-old. Ozbek et al. [16] treated an osteoid osteoma of the proximal phalanx of the fourth finger with RFA, Ramos et al. [13] effectively ablated an osteoid osteoma of the proximal phalanx of the third finger and, more recently, Sangwan et al. [12] successfully treated an OO of the scaphoid with RFA.

Our results were promising. The use of RFA was shown to have an excellent overall complication rate: 25% versus 32% for surgery. The two complications were a local relapse in one patient and a recurrence in another. Both were successfully managed with a second ablation. In contrast, there was no postoperative stiffness or pain (which accounted for 21% of surgical complications) and no heat lesions, proving that correct probe positioning and reduced temperature allow soft tissue not to be injured. Remarkable also was the resumption of work activity in RFA-treated patients, which was faster than the surgical solution.

In agreement with the literature [14], patients with periosteal OO of the hand have not been managed with RFA because they are considered too superficial and therefore destined to have iatrogenic heat injury. This limitation of the technique currently has not yet been overcome but certainly is an interesting topic for future improvements.

This study has some limitations. The small number of patients can create a bias, especially in the complication and recurrence rate. To obtain more reliable data a study with a greater number of patients should be implemented. Ideally, an RCT comparing the two treatment modalities could provide more definitive data on their relative success rate. Another limitation is given by the limitation of RFA to treat periosteal OO, introducing a selection bias.

On the other hand, no previous study has presented a more numerous case series of OO of the hand successfully treated with RFA. We believe that by presenting our work we can encourage more surgeons to work as a team with interventional radiologists to ensure the best possible outcome for patients.

## 5. Conclusions

We believe that osteoid osteoma treatment with RFA for the hand should be an available alternative to surgery as it allows rapid pain relief and a swift return to work.

Overall RFA is minimally invasive, safe, and offers advantages over surgical curettage; therefore, we believe surgery should only be used in cases of diagnostic uncertainty or periosteal localization.

## Figures and Tables

**Table 1:** General characteristics of the group.

Characteristics		*n* (%)
Sex	Male	16 (59.3)
	Female	11 (40.7)
Side	Right	15 (55.6)
	Left	12 (44.4)
Bone	Phalanges	15 (55.6)
	Metacarpal	5 (18.5)
	Carpal	7 (25.9)
Site	Periosteal	7 (25.9)
	Intracortical	12 (44.4)
	Subcortical	8 (29.6)

**Table 2:** Curettage and RFA demographics, complications, and outcomes.

Characteristics		Surgery	RFA	*p*-Value
Sex	Male	8 (42.1)	3 (37.5)	0.824
	Female	11 (57.9)	5 (62.5)	
Age		30.2	31.7	
Work	Heavy	9 (47.4)	4 (50.0)	0.937
	Light	6 (31.6)	2 (25.0)	
	Unemployed	4 (21.0)	2 (25.0)	
Bone	Phalanges	12 (63.2)	3 (37.5)	0.469
	Metacarpal	3 (15.8)	2 (25.0)	
	Carpal	4 (21.0)	3 (37.5)	
Site	Periosteal	7 (36.9)	0	0.031
	Intracortical	8 (42.1)	4 (50.0)	
	Subcortical	4 (21.0)	4 (50.0)	
Size	<5 mm	11 (57.9)	2 (25.0)	0.118
	≥5 mm	8 (42.1)	6 (75.0)	
Complications	Stiffness	1 (5.3)	0	
	Pain	3 (15.8)	0	
	Intraoperative fracture	1 (5.3)	0	
	Local Relapse	1 (5.3)	1 (12.5)	
	Heat Lesions	0	0	
Recurrence		0	1 (12.5)	
Return to work	Early	15 (79.0)	8 (100)	0.160
	Delayed	4 (21.0)	0	

**Table 3:** Median pre-operative and post-operative outcomes and relative *p*-values.

Treatment	Outcome	Pre-Treatment	Post-Treatment	*p*-Value
Surgery	VAS pain	7	2	0.029
	DASH score	40	18	0.024
	PRWE score	56	22	0.026
RFA	VAS pain	8	1	0.022
	DASH score	35	19	0.025
	PRWE score	50	18	0.028

## Data Availability

No new data available.
